# Comparison of the efficacy of combining left bundle branch pacing with either sacubitril/valsartan or enalapril in the treatment of chronic heart failure: A retrospective observational analysis

**DOI:** 10.12669/pjms.40.3.8520

**Published:** 2024

**Authors:** Shengping Wang, Yongsheng Feng, Xiangyang Sun, Hualiang Gou, Yong Guo

**Affiliations:** 1 Shengping Wang, Department of Cardiology, Dazhou Central Hospital,Dazhou, Sichuan Province, 635000, China; 2 Yongsheng Feng, Department of Cardiology, Dazhou Integrated Hospital of Traditional Chinese and Western Medicine,Dazhou, Sichuan Province, 635000, China; 3 Xiangyang Sun, Department of Cardiology, Dazhou Central Hospital,Dazhou, Sichuan Province, 635000, China; 4 Hualiang Gou, Department of Cardiology, Dazhou Central Hospital,Dazhou, Sichuan Province, 635000, China; 5 Yong Guo, Department of Cardiology, Dazhou Central Hospital,Dazhou, Sichuan Province, 635000, China

**Keywords:** Left bundle branch pacing, Sacubitril/Valsartan, Enalapril, Chronic heart failure

## Abstract

**Objective:**

To assess the efficacy of left bundle branch pacing (LBBP) combined with either sacubitril/valsartan or enalapril in the treatment of chronic heart failure (CHF).

**Methods:**

We retrospectively reviewed the records of 138 patients with CHF admitted to Dazhou Central Hospital between June 2020 and June 2022 to extract clinical data. We divided the data into two treatment groups for the analysis: 71 patients received LBBP combined with sacubitril/valsartan treatment (sacubitril/valsartan group), and 67 received LBBP combined with enalapril treatment (enalapril group). The levels of cardiac and cardiopulmonary function indicators, levels of myocardial injury markers, and the scores of the Minnesota Living with Heart Failure Questionnaire (MLHFQ) before and after the treatment were compared between the two groups.

**Results:**

After six months of treatment, patients in the sacubitril/valsartan group had lower myocardial injury markers, higher cardiopulmonary function indicators, and lower MLHFQ scores (*P*<0.05).

**Conclusions:**

In CHF patients, the combination of LBBP with sacubitril/valsartan had a better therapeutic effect compared to LBBP with enalapril, with more effective improvement of the cardiopulmonary function, reduction of myocardial injury, and improvement in quality of life.

## INTRODUCTION

Chronic heart failure (CHF), a common multifactorial cardiovascular disease leading to changes in cardiac function and structure, affects 64.3 million people worldwide.^1^ Long-term exposure to various chemicals and adverse overload factors can decompensate cardiac mechanisms, ultimately leading to heart failure.^1^,^2^ The incidence of CHF has increased in many regions of the world with the aging of the population, and became a social and public health problem that seriously impacts quality of life and physical and mental health of the patients, and is associated with heavy economic and medical burden.^3^,^4^ Thus, early adoption of effective CHF treatment measures is crucial.

Left bundle branch pacing (LBBP) is an important CHF treatment that alleviates clinical symptoms and improves cardiac function of patients.^5^ Current evidence suggest that treatment with certain medications after LBBP can further improve the effectiveness of the intervention ensuring satisfactory disease outcomes.^6^ Angiotensin-converting enzyme inhibitors, including enalapril, are important therapeutic drugs for CHF that are commonly used to inhibit the production of angiotensin II and aldosterone secretion, thereby reducing the cardiac load and improving myocardial remodeling.^7^ Encephalinase inhibitors/angiotensin receptor blockers, such as sacubitril/valsartan, are a new type of anti-heart failure treatment 7 that inhibits both encephalinase and the angiotensin receptor, and helps reverse myocardial remodeling, thus lowering blood pressure, and improving diuresis.^7^,^8^

In recent years, studies have investigated the efficacy of sacubitril/valsartan combined with other medications in patients with CHF, but there are few data on the combination of this drug regimen with LBBP.^9^,^10^ Therefore, we conducted a retrospective analysis of the clinical data of patients with CHF who received LBBP combined with enalapril or sacubitril/valsartan treatment in our hospital, with the aim of clarifying their specific intervention outcomes. We believe that our results may be useful as a reference for caregivers to select optimal treatment plans for their patients with CHF. The objective of this study was to assess the efficacy of LBBP combined with either sacubitril/valsartan or enalapril in the treatment of CHF.

## METHODS

In this retrospective observational analysis, we reviewed the clinical data from 138 patients with CHF admitted to Dazhou Central Hospital from June 2020 to June 2022. According to the clinical records, 71 patients received LBBP combined with sacubitril/valsartan (sacubitril/valsartan group) and 67 patients received LBBP combined with enalapril (enalapril group).

### Ethical Approval

The ethics committee of Dazhou Central Hospital approved this study with the number 2023-028, April 24^th^, 2023.

### Inclusion criteria:

- Patients meeting the diagnostic criteria for CHF.^11^
- Patients with left ventricular ejection fraction (LVEF) ≤35%.
- Patients with left ventricular end diastolic diameter (LVEDD) ≥60 mm.
- Presence of intraventricular or atrioventricular block.
- Patients with clinical diagnosis and treatment results and other complete information.
- Patients with New York Heart Association (NYHA) grading for cardiac function-II to III.

### Exclusion criteria:

- Patients with angina pectoris or acute myocardial infarction.
- Patients with severe damage to kidney or liver functions.
- Lactating or pregnant women.
- Patients with acute or chronic infectious diseases.
- Patients with vascular or nerve edema.

### Treatment methods

Patients in both groups received LBBP. For patients who could not take β-blockers due to atrioventricular block before LBBP, metoprolol was administered after LBBP. Patients in the sacubitril/valsartan and enalapril groups received sacubitril/valsartan and enalapril, respectively, in combination with the LBBP. Patients in both groups were observed for six months. The LBBP procedure and the protocol of sacubitril/valsartan and enalapril treatment were as follows”

1) LBBP procedure - A physician gained access through the axillary vein and inserted an active fixed wire (Medtronic Select Secure 3830 electrode) through the C315 Hester bundle sheath tube guided by X-ray fluoroscopy in a 30° right anterior oblique position. At 1.5-2 cm of the interventricular septum, at the far end of the His bundle, the QRS wave in lead V1 showed a “W” shape during pacing. After rotating counterclockwise to ensure that the sheath tube was perpendicular to the lead and the ventricular septum, the physician slowly rotated the lead (3830 electrode) into the left bundle branch area under the left interventricular septum intima. During the insertion of lead (3830 electrode), the QRS notch of lead V1 shifted backwards, and the QRS wave showed a QR shape in the later stage of insertion. The left bundle branch range was reached and the pacing QRS wave showed a right bundle branch block-like feature (rSr type). In some patients the left bundle branch potential was recorded approximately 20 minutes ahead of the start of the QRS wave on the body surface. The time from the V5 lead pacing nail to the peak of the R wave was recorded.
2) Enalapril tablets (Jiangsu Pharmaceutical Co., Ltd., 5mg/tablet, H32026568) - Patients were initially administered 5mg tablets once a day, the dose was gradually adjusted to two tablets a day on the basis of blood pressure status.
3) Sacubitril/valsartan sodium tablets (Beijing Novartis Pharmaceutical Co., Ltd., 100 mg, J20171054) - Patients were initially prescribed 25mg, twice a day, and the dose was gradually adjusted to the maximum tolerated dose (75mg, twice a day) according to blood pressure, which was no lower than 90/60 mmHg.

The following patient baseline data and relevant indicators were collected before and six months after treatment:

### Cardiac function indicators

The levels of left ventricular end-systolic diameter (LVESD), left ventricular end-diastolic diameter (LVEDD), left ventricular ejection fraction (LVEF), and left ventricular mass index (LVMI) were collected. They were measured using a cardiac ultrasound diagnostic instrument (Mindray Company, Shenzhen, DC-8EXP).

### Myocardial injury markers

The levels of soluble suppression of tumorigenicity2 (sST2) and N-terminal pro B-type natriuretic peptides (NT proBNP) were collected. Blood samples were taken by the patients and were centrifuged at 3500 r/min for 10 min to extract the serum. sST2 was determined using an enzyme-linked immunosorbent assay, and NT proBNP was tested using a fluorescein-enhanced immunochemiluminescence assay. All reagent kits were purchased from Shanghai Mlbio Biotechnology and assays were performed strictly according to the manufacturer’s instructions.

### Cardiopulmonary function indicators

The levels of maximum exercise time, maximum exercise power, and peak oxygen consumption (peak VO_2_) were measured using the Swiss Schiller cardiopulmonary exercise testing system (SCHILIER-CS200).

### Quality of Life (QoL)

QoL was assessed using the Minnesota Heart Failure Quality of Life Questionnaire (MLHFQ). MLHFQ data is routinely collected by the nurses of our center for all patients with their consent. MLHFQ has a total of 105 points. Lower score correlates with the higher quality of life.^12^

### Statistical analysis

All data analysis was conducted using SPSS v24.0 (SPSS, Chicago, IL, USA). The normality of the data was evaluated using the Shapiro-Wilk test. The data of normal distribution were expressed by mean ± standard deviation, and the inter group comparison was performed by independent sample t test. Paired t-tests were used for intra group comparison before and after the treatment. Non-normal distribution data were expressed by median and interquartile range, and Mann-Whitney U test was used for inter-group comparison; The Wilcoxon signed rank test was used for intra group comparison before and after the treatment, based on the obtained clinical records of the patients. The counting data are represented by the number of use cases, and Chi-squared test is used. When P<0.05, the difference is considered statistically significant.

## RESULTS

A total of 138 patients (77 men and 61 women) were included in this study. Patients were aged 54 to 84 years (average, 70.14±6.00 years). There were 82 cases of NYHA grade II and 56 patients with grade III. The disease course ranged from one to nine years, with a median course of 5 (3, 6) years. In terms of primary disease types, 71 patients presented dilated cardiomyopathy and 67-ischemic cardiomyopathy.

The sacubitril/valsartan group consisted of 38 men and 33 women with an age range between 54 and 82 years (average, 69.63±5.98 years); NYHA classification of cardiac function grades II (n=41) and III (n=30); Disease course of one to nine years, with a median course of 5 (4,6) years. Of 71 patients in the group, 39 had dilated cardiomyopathy and 32 had ischemic cardiomyopathy. The enalapril group consisted of 39 men and 28 women with an age range between 58 and 84 years (average, 70.69±6.02 years); NYHA classification of cardiac function grades II (n=41) and III (n=26); Disease course of one to eight years, with a median course of 5 (3,5) years. There were 32 cases of dilated cardiomyopathy and 35 cases of ischemic cardiomyopathy in this group. All baseline variables were comparable in the two groups (P>0.05) (Table-I).

**Table-I:** Patient characteristics

Group	n	Gender (male/female)	Age (years)	NYHA classification (Grade II/ Grade III)	Course of diseases (year)
Sacubitril/valsartan group	71	38/33	69.63±5.98	41/30	5 (4,6)
Enalapril group	67	39/28	70.69±6.02	41/26	5 (3,5)
*χ^2^*/*t/Z*		0.307	-1.030	0.170	-1.125
*P*		0.579	0.305	0.680	0.261

We also found similar cardiac function indicator values in the two groups before the treatment (P>0.05). After six months of the treatment, the values for LVESD, LVEDD, and LVMI in the two groups decreased compared to the baseline values, whereas the LVEF values increased compared to the corresponding baseline values (P<0.05). Additionally, the mean LVESD, LVEDD, and LVMI were lower, and the mean LVEF was higher in the sacubitril/valsartan group compared to the enalapril group (P<0.05) (Table-II). Similar levels of myocardial injury markers were found in the two groups before the treatment (P>0.05). After six months of the treatment, the mean blood levels of sST2 and NT proBNP in the two groups decreased compared to the baseline values, and these levels were lower in the sacubitril/valsartan group (P<0.05) (Table-III).

**Table-II:** Comparison of cardiac function indicators between two groups.

Time	Group	n	LVESD (mm)	LVEDD (mm)	LVEF (%)	LVMI (g/m^2^)
Before treatment	Sacubitril/valsartan group	71	58(52, 63)	68(62, 70)	32(29, 34)	136.15±6.61
	Enalapril group	67	59(53, 63)	68(63, 71)	31(28, 33)	137.30±6.29
	t/Z		-1.123	-0.926	-0.919	-1.040
	P		0.261	0.354	0.358	0.300
After treatment	Sacubitril/valsartan group	71	51(46, 55)^a^	60(55, 63)^a^	51.74±4.91^a^	120.41±5.87^a^
	Enalapril group	67	55(52, 57)^a^	66(62, 69)^a^	45.37±4.86^a^	124.76±5.00^a^
	t/Z		-4.859	-6.522	7.660	-4.674
	P		<0.001	<0.001	<0.001	<0.001

***Note:*** Compared with before treatment in this group,

a P<0.05.

**Table-III:** Comparison of levels of myocardial injury markers between two groups.

Time	Group	n	sST2 (μg/L)	NT-proBNP (μg/L)
Before treatment	Sacubitril/valsartan group	71	0.71(0.51, 0.87)	6589(6158, 7247)
	Enalapril group	67	0.66(0.48, 0.85)	6478(6025, 7036)
	Z		-0.846	-1.025
	P		0.397	0.306
After treatment	Sacubitril/valsartan group	71	0.34(0.19, 0.50)^a^	1497(1124, 1659)^a^
	Enalapril group	67	0.44(0.25, 0.63)^a^	1972(1656, 2423)^a^
	Z		-3.196	-7.368
	P		0.001	<0.001

***Note:*** Compared with before treatment in this group,

a P<0.05.

Similar cardiopulmonary function indicators were found in the two groups before the treatment (P>0.05). After six months of the treatment, the maximum exercise time, maximum exercise power, and peak VO_2_ values increased in the two groups compared to the baseline values and were significantly higher in the sacubitril/valsartan group (P<0.05) (Table-IV).

**Table-IV:** Comparison of cardiopulmonary function indicators between two groups.

Time	Group	n	Maximum exercise time (minute)	Maximum motion power (W)	peak VO_2_(ml/min^-1^·kg^-1^)
Before treatment	Sacubitril/valsartan group	71	6.45±1.35	101(89, 116)	17(15, 18)
	Enalapril group	67	6.27±1.31	102(95, 115)	18(16, 19)
	t/Z		0.841	-0.829	-1.430
	P		0.402	0.407	0.153
After treatment	Sacubitril/valsartan group	71	8.83±1.46^a^	126(118, 141)^a^	23(21, 24)^a^
	Enalapril group	67	7.18±1.39^a^	115(108, 128)^a^	20(18, 21)^a^
	t/Z		6.815	-4.454	-7.488
	P		<0.001	<0.001	<0.001

There were no significant differences in MLHFQ scores between the two groups before the treatment (P>0.05). After six months of treatment, the MLHFQ scores in the two groups decreased compared to the corresponding baseline values, and the MLHFQ scores of the sacubitril/valsartan group were lower (P<0.05) (Table-V).

**Table-V:** Comparison of Quality of Life Scores between Two Groups.

Group	n	Before treatment	After treatment	Z	P
Sacubitril/valsartan group	71	47(44, 50)	39(34, 42)	-7.866	<0.001
Enalapril group	67	48(43, 51)	43(38, 46)	-7.836	<0.001
Z		-1.024	-4.514		
P		0.306	<0.001		

## DISCUSSION

The results of this study indicated that in the treatment of CHF, the combination of LBBP and sacubitril/valsartan can more effectively improve cardiac and pulmonary functions, reduce the degree of myocardial injury, and is more conducive to improving the quality of life of patients than the combination of LBBP with enalapril.

Enalapril and sacubitril/valsartan are commonly used in the clinical treatment of CHF, and the differences in their effects have been explored. Kang H et al^13^ compared sacubitril/valsartan with other drugs such as enalapril, valsartan, and irbesartan in a meta-analysis, and showed that sacubitril/valsartan has significant advantages for improving the glomerular filtration rate and downregulating NT-proBNP levels. Our observations are consistent with these results. The sacubitril/valsartan combination that we tested is the first developed angiotensin receptor encephalinase inhibitor that can simultaneously inhibit both the angiotensin II receptor and the encephalinase.^8^,^13^,^14^ In addition, sacubitril/valsartan has two targets of action. As valsartan can inhibit the renin–angiotensin–aldosterone system (RAAS),^13^,^14^ sacubitril/valsartan can be metabolized by liver enzymes into the active encephalinase inhibitor LBQ657, which inhibits the growth of encephalinase enzymes, reduces the degradation of natriuretic peptide, upregulates the content of natriuretic peptide, improves nitric oxide bioavailability, dilates blood vessels, reduces the cardiac load;^8^,^14^ and, prevents the release of aldosterone and renin, and myocardial hypertrophy, thus improving ventricular remodeling.^13^-^15^

Tsutsui H et al.^16^ confirmed that both sacubitril/valsartan and enalapril can reduce cardiovascular adverse event mortality and heart failure readmission rates in patients with CHF, but sacubitril/valsartan has superior safety and tolerance profiles. Moreover, sacubitril/valsartan has a dual mechanism of action and belongs to a new type of specific salt complex crystal formulation^15^,^16^ that inhibits the RAAS and regulates the natriuretic peptide system, thereby delaying or reversing ventricular remodeling and achieving the goal of improving cardiac function.^14^,^16^ Pieske et al.^17^ found that sacubitril/valsartan can inhibit the vasodilation, diuresis, and natriuretic effects of encephalinase and RAAS, effectively improving cardiac function and long-term prognosis in patients with CHF. Moreover, sacubitril/valsartan can reduce cellular responses, fibrosis, and inflammation mediated by aldosterone, mineralocorticoid receptors, and angiotensin AT1 receptors, thereby alleviating vascular, cardiac, and renal damage.^18^,^19^

In addition, our study showed that the mean improvements in myocardial injury marker levels in the sacubitril/valsartan group were superior to those in the enalapril group. NT proBNP is an inactive terminal metabolite formed after brain natriuretic peptide division. It is an important marker of heart failure and its half-life is longer than that of BNP.^20^ sST2, a member of the interleukin-1 receptor family, can antagonize myocardial remodeling, inhibit myocardial hypertrophy, and protect cardiac function. Therefore, both sST2 and NT proBNP can accurately and objectively reflect the degree of cardiac function damage and prognosis in CHF patients.^21^

CHF is associated with frequent rehospitalizations and poor quality of life.^22^ A recent systematic review and meta-analysis of randomized controlled trials showed that sacubitril/valsartan is beneficial to improve health-related quality of life in patients with CHF.^23^ Consistently, the results of our study also show that sacubitril/valsartan can better ensure the quality of life of CHF patients after the treatment.

### Limitations

Current study has a small sample size and only analyzed records from one hospital. We did not detect any adverse reactions in the patients and did not conduct long-term follow-ups. The prognosis of the patients remained unclear. Further large-scale, multicenter, and long-term randomized controlled trials are needed to validate our results.

## CONCLUSION

The combination of LBBP with sacubitril/valsartan showed a better therapeutic effect in CHF patients than LBBP with enalapril. Combined LBBP and sacubitril/valsartan treatment was more effective in improving patients’ cardiopulmonary function, reducing myocardial injury, and was associated with the improvement in patients’ quality of life.

### Authors’ contributions:

**SW:** Conceived and designed the study.

**YF**, **XS**, **HG** and **YG:** Collected the data and performed the analysis.

**SW:** Was involved in the writing of the manuscript and is responsible for the integrity of the study.

All authors have read and approved the final manuscript.
